# Classification of the renal papillary abnormalities by flexible ureteroscopy: evaluation of the 2016 version and update

**DOI:** 10.1007/s00345-020-03149-4

**Published:** 2020-03-19

**Authors:** Christophe Almeras, Michel Daudon, Vincent Estrade, Jean Romain Gautier, Olivier Traxer, Paul Meria

**Affiliations:** 1 Department of Urology, La Croix du Sud Clinic, 52 chemin de Ribaute, 31130 Quint Fonsegrives, France; 2 Unit of Functional Explorations, Tenon Hospital, Pierre and Marie Curie University, Paris, France; 3 Department of Urology, Hospital, Angoulême, France; 4 Department of Urology, Tenon Hospital, Pierre and Marie Curie University, Paris, France; 5 Department of Urology, Saint-Louis Hospital, Denis Diderot University, Paris, France

**Keywords:** Kidney, Stone, Renal, Papillary abnormalities, Classification

## Abstract

**Introduction:**

To assess the use of the 2016 proposed classification of the renal papillary abnormalities during flexible ureteroscopy that aims to standardize their description.

**Patients and methods:**

We performed a prospective monocentric single operator collection of the data using this classification during 88 consecutive flexible ureteroscopies required for renal stones treatment. Outcome measurements and statistical analysis: data of stones analysis (microscopy and infrared spectrophotometry) and of serum and urines biochemical samples have been compared with the results of the classified endoscopic descriptions.

**Results:**

Mean duration of description was 81.4 s. We reported that 83% of the patients had Randall plaques (RP), as only 4.5% of the patients had no abnormality. Concerning the papillary stones and anchored stones were observed in 30.7% and aspect of intraductal crystallization (Sc) in 15.9%. Erosions were present in 55.7% and extrophic papillae in 8%. Sa1 and Pa2 were significantly correlated to RP, anchored stones (Sa) to papillary erosions and calcium phosphate stones to intraductal crystallization. Hypercalciuria was significantly higher in Sa2 than Sa1 stones.

**Conclusions:**

The different descriptions in the 2016 classification were confirmed by the results of this study. Papillary abnormalities are consequences of stones development. Their descriptions could also improve the follow-up and the diagnosis of a metabolic lithogenesis. We recommend their systematic description during ureteroscopy. Some improvements are proposed to update this classification.

## Introduction

Since Randall in the 1930s [1], the fact that papillary calculi resulted from subepithelial lesions is well known. Many papillary abnormalities can appear in the first steps of stone disease formation, even during the long-term evolution of stone disease. The first endoscopic descriptions concerned mainly the presence of Randall’s plaques (RP) [2], nonetheless many other abnormalities could be observed during flexible ureteroscopy leading to the elaboration of a severity grading score [3] and then a classification in 2016 [4].

**Fig. 1:**
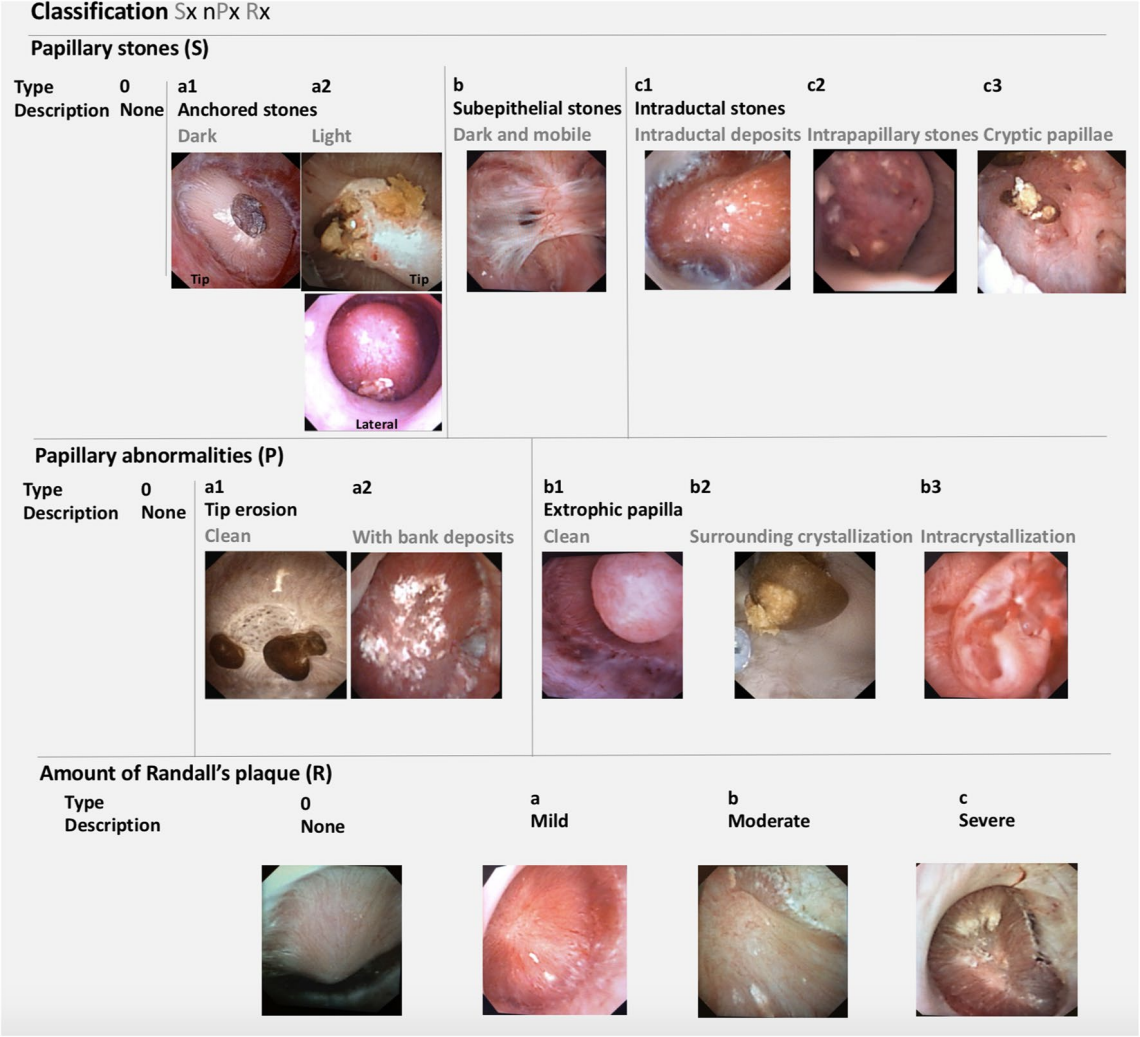
Sx nPx Rx classification (2016): *S* represents the type of papillary stone observed, *n* the number of papillae in the same kidney concerned by the type of observed abnormality (P) and *R* the amount of Randall’s plaques

As papillary stones and papillary abnormalities can be associated in the same patient, and inspired by the oncologic TNM classification, the 2016 endoscopic classification included a stone description (Sx), the number and type of papillary abnormality observed (nPx), and the amount of Randall’s plaque (Rx). This description (confirmed by the different authors) resumed the abnormalities of all the papillae in the same kidney. For example, in a patient with anchored dark calculi and erosions with bank deposits on 4 papillae with severe amount of Randall’s plaque, the stage was: Sa1 4Pa2 Rc (Fig. 1) [4]. This classification seems to be complex at first sight, but was at this time the only way to describe the associated abnormalities. The grading score as the endoscopic classification suggested a potential major impact in predicting recurrence and in understanding the pathogenesis of nephrolithiasis for a stone former. The aim of this study was to evaluate the 2016 classification after systematic use.

## Patients and methods

We performed a prospective monocentric single operator study. Data were prospectively collected during consecutive flexible ureterorenoscopies required for the treatment of renal stones from May 2016 to March 2019. Karl Storz flexible digital ureterorenoscopes were used during the procedures (digital 11278VK). Interventions requiring a fiberoptic ureteroscope during the periods of unavailability of the digital device and ureteroscopies combined with percutaneous nephrolithotomies have been excluded from this study because of not allowing an optimal endoscopic exploration of the kidney. A systematic endoscopic exploration of all the calyces of the ipsilateral kidney has been made prior to the stone treatment. The exploration began from the upper to the lower calyx, observing systematically all the papillae. The exploration has been recorded systematically and duration has been noticed. Every patient had been classified (one score per kidney and patient) during the procedure at the end of the exploration by the operator (who was blinded to any information in case of prior analysis results of stone, blood, and urine samples) using the Sx nPx Rx classification published by Almeras [4] (Fig. 1). The use of this classification needed to identify the most severe abnormality (potentially correlated with a metabolic lithogenesis) when many abnormalities were associated in the same kidney. Papillary calculi were systematically treated using basket stone extractor (Dakota (Boston Scientifics)) or Holmium laser pulverization (Lumenis Edap). To avoid bleeding and papillary injury, no biopsy has been made, even Kuo reported the feasibility of the endoscopic renal papillary biopsies in 2003 [5]. The collected stones were examined by microscopy and infrared spectrometry (Perlin Elmer) and classified according to the Michel Daudon’s classification [6]. Patients systematically underwent biochemical investigations (serum analysis, 24 h urine analysis) at least one month after the surgical procedure.

The primary endpoint was to describe the results of the different observations and their statistical relations using the Sx nPx Rx classification correlated to stone analysis and the types of lithogenesis.

## Statistics

Categorical variables were analyzed using chi-square test or Fisher’s exact test as appropriate, and continuous variables were analyzed using Student’s *t*-test. The limit of statistical significance was defined as p < 0.05.

## Results

Eighty-eight procedures had inclusion criteria and usable collected data. The features of the population were 65 and 35% of men and women, with a mean age of 55 years (25–98). The mean duration of exploration of the kidney was 81.4 s (range: 48–149; median 64).

The results of the observation of the abnormalities of the papillae were resumed in Table 1. The correlation between the amount of RP and the Sx Px descriptions was reported in Table 2. The results of the correlations between the stone composition and the Sx description were reported in Table 3.

**Table 1:** Repartition of the described papillary abnormalities, according to the Sx nPx Rx 2016 Almeras classification

Abnormalities	*n*	%
Sa1	16	18.2
Sa2	11	12.5
Anchored stones	27	30.7
Sc1	2	2.3
Sc2	9	10.2
Sc3	3	3.4
Intraductal stones	14	15.9
Pa1	23	26.1
Pa2	26	29.5
Papillary erosions	49	55.7
Pb1	6	6.8
Pb2	1	1.1
Pb3	0	0.0
Extrophic papillae	7	8.0
Ra	34	38.6
Rb	26	29.5
Rc	13	14.8
Randall’s plaques	73	83.0
S0	47	53.4
P0	29	33.0
R0	12	13.6
S0 P0 R0	4	4.5
S0 P0 R+	13	14.8

**Table 2:** Correlation between the amount of RP and the Sx Px descriptions according to the 2016 Almeras classification

		*n*	%
S0	R0-a	28	62.2
	Rb-c	17	37.8
Sa1	R0-a	3	18.8
	Rb-c	13	81.3
Sa2	R0-a	6	66.7
	Rb-c	3	33.3
Sa	R0-a	9	36.0
	Rb-c	16	64.0
Sc	R0-a	8	57.1
	Rb-c	6	42.9
P0	R0-a	18	66.7
	Rb-c	9	33.3
Pa1	R0-a	12	57.1
	Rb-c	9	42.9
Pa2	R0-a	11	42.3
	Rb-c	15	57.7
Pa	R0-a	23	48.9
	Rb-c	24	51.1
Pb	R0-a	4	57.1
	Rb-c	3	42.9

**Table 3:** Correlations between the stone’s composition and the Sx description according to the classifications of Daudon and Almeras

Stones	*n*	%/ stones	S0	%	Sa1	%	Sa2	%	Sc	%
Ia	39	48.8	21	50.0	12	75.0	3	27.3	4	28.6
Ib	3	3.8	2	4.8	0	0.0	1	9.1	0	0.0
Ic	2	2.5	0	0.0	0	0.0	0	0.0	2	14.3
Id	1	1.3	1	2.4	0	0.0	1	9.1	0	0.0
Ie	1	1.3	0	0.0	0	0.0	1	9.1	0	0.0
COM	46	57.5	24	57.1	12	75.0	6	54.5	6	42.9
IIa	5	6.3	2	4.8	0	0.0	1	9.1	2	14.3
IIb	5	6.3	3	7.1	0	0.0	2	18.2	0	0.0
COD	10	12.5	5	11.9	0	0.0	3	27.3	2	14.3
IIIa	7	8.8	3	7.1	3	18.8	0	0.0	1	7.1
IIIb	6	7.5	5	11.9	0	0.0	0	0.0	1	7.1
UA	13	16.3	8	19.0	3	18.8	0	0.0	2	14.3
IVa1	5	6.3	2	4.8	1	6.3	1	9.1	1	7.1
IVa2	2	2.5	0	0.0	0	0.0	1	9.1	2	14.3
IVd	1	1.3	0	0.0	0	0.0	0	0.0	1	7.1
CP	8	10.0	2	4.8	1	6.3	2	18.2	4	28.6
Struvite	0	0.0	0	0.0	0	0.0	0	0.0	0	0.0
Va	3	3.8	3	7.1	0	0.0	0	0.0	0	0.0
			42		16		11		14	

In this table, the intraductal crystallization subgroups Sc1, Sc2, and Sc3 have been combined in one single group (Sc) to enhance the power of description

COM, calcium oxalate monohydrate (I); COD, calcium oxalate dihydrate (II); UA, Uric acid (III); CP, calcium phosphate (IV)

Statistical analysis revealed a significant correlation between a high amount of Randall plaques with the presence of anchored dark stones Sa1 (p = 1.95 10^–5^), and with the description of papillary erosions with bank deposits Pa2 (*p* = 0.0011). A high amount of RP was also significantly more observed in case of the presence of dark anchored stones (Sa1) than in case of intraductal crystallization (Sc) (*p* = 0.035).

The presence of papillary erosions (Pa) was significantly correlated to the presence of anchored stones (Sa) (*p* = 0.016), and significantly more observed in case of anchored stones (Sa) than in case of intraductal crystallization (Sc) (*p* = 0.0041).

The calcium phosphate (CP) stones and especially the IVa2 type were significantly more frequent in case of intraductal crystallization (Sc) (*p* = 0.023 and 0.028). Intraductal crystallization (Sc) had also a significant different repartition of the types of stones (*p* = 0.039) in comparison to the other papillary stones.

Only 69 patients had available biochemical investigations. The results (Table 4) reported that an hypercalciuria was significantly more diagnosed with Sa2 than Sa1 anchored stones (45.5 vs 7.7%; *p* = 0.048). Urinary tract infection was mainly correlated to few papillary abnormalities, and intraductal crystallization to hypocitraturia (55.6%) and hypercalciuria (33.3%).

**Table 4:** Results of the biochemical investigations and correlation (%) with the papillary abnormalities (2016 classification)

Abnormalities	Hyper Ca	HyperPTH	Hyper Ox	Hyper U	Hypocit	HypoMg	Low fluid intake	Hyper Na	HyperAzot	Diabetes	UTI
Sa1	7.7	0	0	23.1	15.4	23.1	53.8	30.8	38.5	0	0
Sa2	45.5	9.1	0	27.3	27.3	9.1	36.4	27.3	18.2	0	0
Anchored s.	25	4.2	0	25.0	20.8	16.7	45.8	29.2	29.2	0	0
Sc1	50	50	0	0	50	0	0	0	0	0	0
Sc2	40	0	20	40	60	20	40	0	0	20	16.7
Sc3	0	0	50	0	50	50	50	50	0	0	0
Intraductal s.	33.3	11.1	22.2	22.2	55.6	22.2	33.3	11.1	0	11.1	10
Pa1	21.1	0	10.5	21.1	26.3	26.3	31.6	31.6	21.1	5.3	9.5
Pa2	13.6	11.5	0	23.1	30.8	34.6	42.3	11.5	19.2	7.7	3.8
Erosions	17.1	7.3	4.9	24.4	31.7	34.1	41.5	22.0	22	7.3	7
Pb1	60	0	20	40	20	20	60	0	0	0	0
Pb2	100	0	0	0	0	0	0	100	0	0	0
Pb3	0	0	0	0	0	0	0	0	0	0	0
Extrophic p.	66.7	0	16.7	33.3	16.7	16.7	50	16.7	0	0	0
Ra	28.6	7.1	7.1	25	25	17.9	39.3	21.4	7.1	7.1	6.7
Rb	8.3	8.3	4.2	20.8	25	29.2	41.7	20.8	37.5	8.3	12.5
Rc	28.6	0	0	14.3	28.6	57.1	28.6	14.3	14.3	0	0
Randall Pl.	20.3	6.8	5.1	22	25.4	27.1	39	20.3	20.3	6.8	8.2
S0	8.3	5.6	5.6	19.4	19.4	38.9	30.6	16.7	13.9	13.9	21.1
P0	5	5	5	15	15	25	25	20	15	15	28.6
R0	0	0	12.5	25	25	50	25	25	0	25	44.4

HyperCa, hypercalciuria; HyperPTH, hyperparathryroidism; HyperOx, hyperoxaluria; HyperU, hyperuricuria; Hypocit, hypocitraturia; HypoMg, hypomagnesuria; HyperNa, hypernatriuria; HyperAzot, hyperazoturia; UTI, urinary tract infection

## Discussion

The fact that papillary calculi result from subepithelial lesions is well known since Randall in the 1930s [1]. Most lesions of RP undergo calcification by carbapatite (CA) and could be removed by macrophages recruited by proteins as osteopontin binded to hydroxyapatite (HAP) [7]. Recent studies based on the kidney biopsies evidenced that apatite deposits at the origin of these plaques originate from the basement membranes of thin loops of Henle, especially in the ascending thin limbs [8] and then spread in the surrounding interstitium around descending vasa recta [9, 10]. These deposits grow and erode the epithelium covering the papillae, resulting in stone development by the contact with urines. The calcium oxalate monohydrate (COM) crystals are the first one (after urinary apatite), due to their small size, able to combine with HAP [9]. Nonetheless, stone formation on RP is not the only cause of crystallization. Plugs of CA in the Bellini ducts have been also reported on biopsies, with a high risk of interstitial fibrosis surrounding these ducts [10]. In this case, a high incidence of CA stones type IVa2 has been observed especially when plugs were described [4] that should evoke a potential risk of renal tubular acidosis (DTA) and nephrocalcinosis [11]. In our study, hypercalciuria and hypocitraturia were observed in 33.3 and 55.6% of the intraductal crystallization endoscopic abnormalities that could suggest a DTA indeed. Brushite overgrowth on ductal apatite plugs has been also observed by Williams [12], as a mechanism of early intraductal stone growth and retention, confirming the importance and the gravity of this description in care for stone formers. Intraductal crystallization was also responsible for early recurrence in many patients of our study caused by the intraductal stones that were not treated during the surgical procedure and a potential severe underlying metabolic trouble.

In 1997, Low [2] studied the endoscopic mapping of Randall’s plaques. They were found in 74% in patients with stone disease vs 43% without stone disease. In 2006, Matlaga described by endoscopy that 91% of the papillae contained Randall’s plaque, with a prevalence of 48% of attached stones in calcium oxalate stone formers [13]. In 2015, Borofsky [3] proposed a grading system to describe the endoscopic renal papillary appearance in patients with nephrolithiasis. This grading scale described the severity of the abnormalities of one papilla based on plugging (anchored calculi), pitting (tip papillary erosion), loss of contour, and amount of RP. In 2016, the first classification (Sx nPx Rx) describing the severity and the type of papillary abnormalities was published, distinguishing the crystallization on RP and the intraductal crystallization (with a ratio of 77 and 13.5%) [4]. In 2017, Cohen [14] demonstrated the correlation between the presence of tip papillary erosions (pits) and RP, confirming the potential etiologic interest of the knowledge of the papillary abnormalities.

In our study, using de Sx nPx Rx 2016 classification, we reported that 83% of the papillae of stone formers had RP, as only 4.5% of the papillae were “normal” (S0 P0 R0). Concerning the description of papillary stones, anchored stones (Sa) were observed in 30.7% and an aspect of intraductal crystallization (Sc) in 15.9%. Erosions were present in 55.7% and extrophic papillae in 8%. Sa1 and Pa2 were significantly correlated to RP, anchored stones (Sa) to papillary erosions, and CP stones to intraductal crystallization. As expected, hypercalciuria was significantly higher in Sa2 than in Sa1 COM stones.

**Fig. 2:**
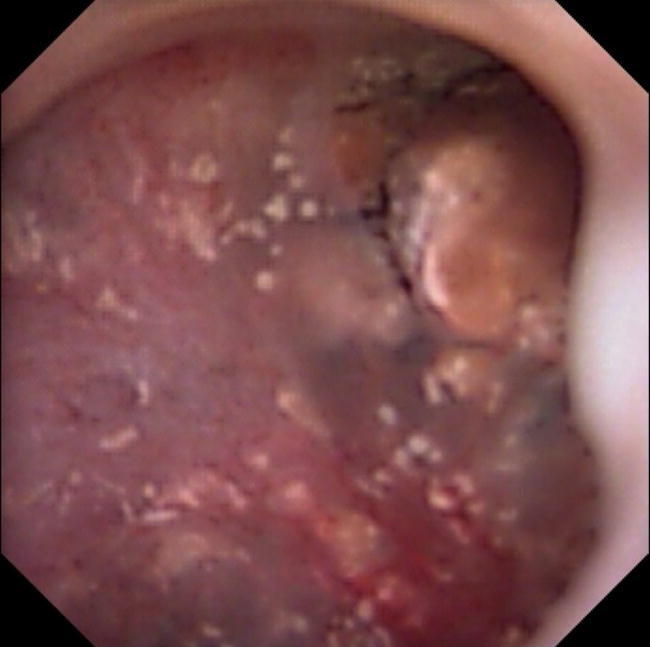
Intraductal plugs (Dp)

**Fig. 3:**
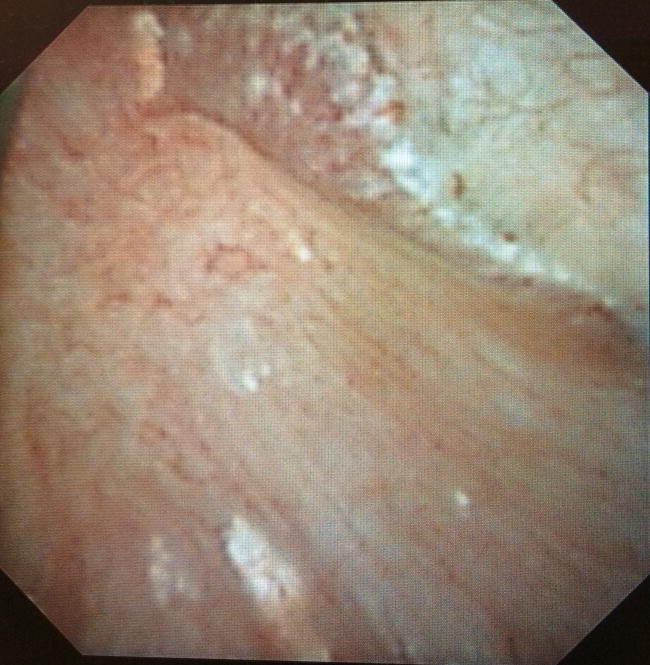
Randall’s plaque (Dr)

**Fig. 4:**
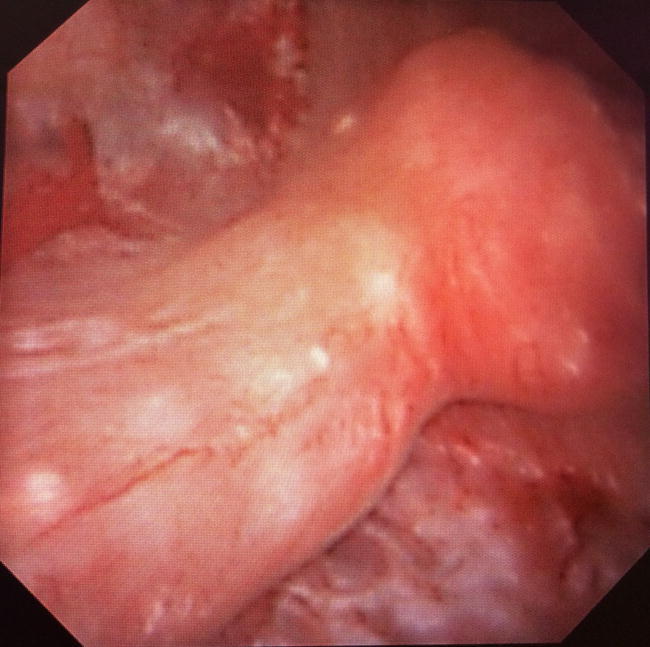
Active intrapapillary popular deposits (Di)

**Fig. 5:**
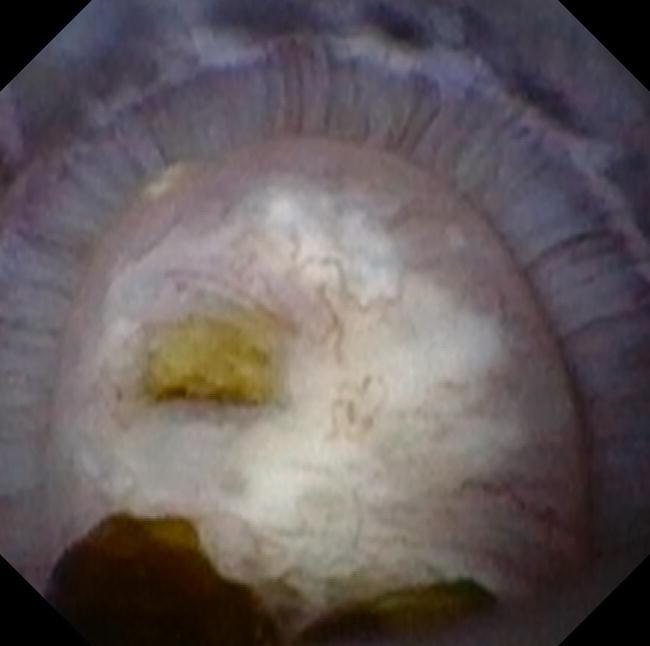
Solitary ischemic extrophic papilla, with active lithogenesis (Pb2)

The systematic description of the papillary abnormalities allowed the demonstration that different groups of abnormalities were related to different lithogenesis mechanisms. Our results confirm statistically the recent findings of Borofsky [15]. The observation of pits or erosion (induced by stone drop-off) and anchored stones were indeed related to calcium oxalate lithogenesis on RP, whereas intraductal stones and cryptic papillae were related to intraductal crystallization on plugs. A lack of accuracy and some difficulties were sometimes encountered by determining if some stones were “RP-anchored” or “plug-anchored”. In order to solve this problem, these stones have to be removed with a basket to examine the papilla and note if there were plugs beneath. The aspect of the plugs (Fig. 2) profoundly differs from the aspect of the RP (Fig. 3). A new entity of deposits has been also described, with an intrapapillary papular ischemic aspect, related to potential active lithogenesis (Fig. 4). The endoscopic recognition of the stones [16] should be also helpful in this case, as there were much more CA stones induced by the intratubular crystallization. The lithogenesis mechanism on extrophic papillae remains unclear and less frequent with a peripheral or an internal crystallization. As only solitary extrophic papilla with an ischemic aspect should be likely to give active lithogenesis (Fig. 5), and as Tanriverdi [17] reported hyperoxaluria-induced tubular ischemia, local ischemia could represent one of the possible etiologic factors of this papillary abnormality.

However, our study had some limitations. The main limitation was certainly the self and prospective evaluation by a single surgeon (scant number of cases and potential self-evaluation bias). The use of this classification by urologists probably needs also a learning curve that requires an assessment of reproducibility and an evaluation in a wider urologic community. The comparison of the score obtained in the same patient (using video recordings) between different urologists should also be the subject of an upcoming study. The small number of cases could also be responsible for no significant drawn conclusion on the biochemical investigations except concerning the observed correlation of the Sa2 anchored stones and the presence of hypercalciuria. A long-term study should be suitable to assess more precisely its impact in predicting the risk of recurrence of stones.

Despite these limitations, these results proved the interest of this classification, describing the type (dark or light) and the location of stones, the type of papillary abnormalities (erosions, cryptic papilla, …), and the amount of RP. It allows also to determine the severity of the lesions and the potential risk of recurrence and to distinguish the type of crystallization (on RP or on intraductal plugs).

**Fig. 6:**
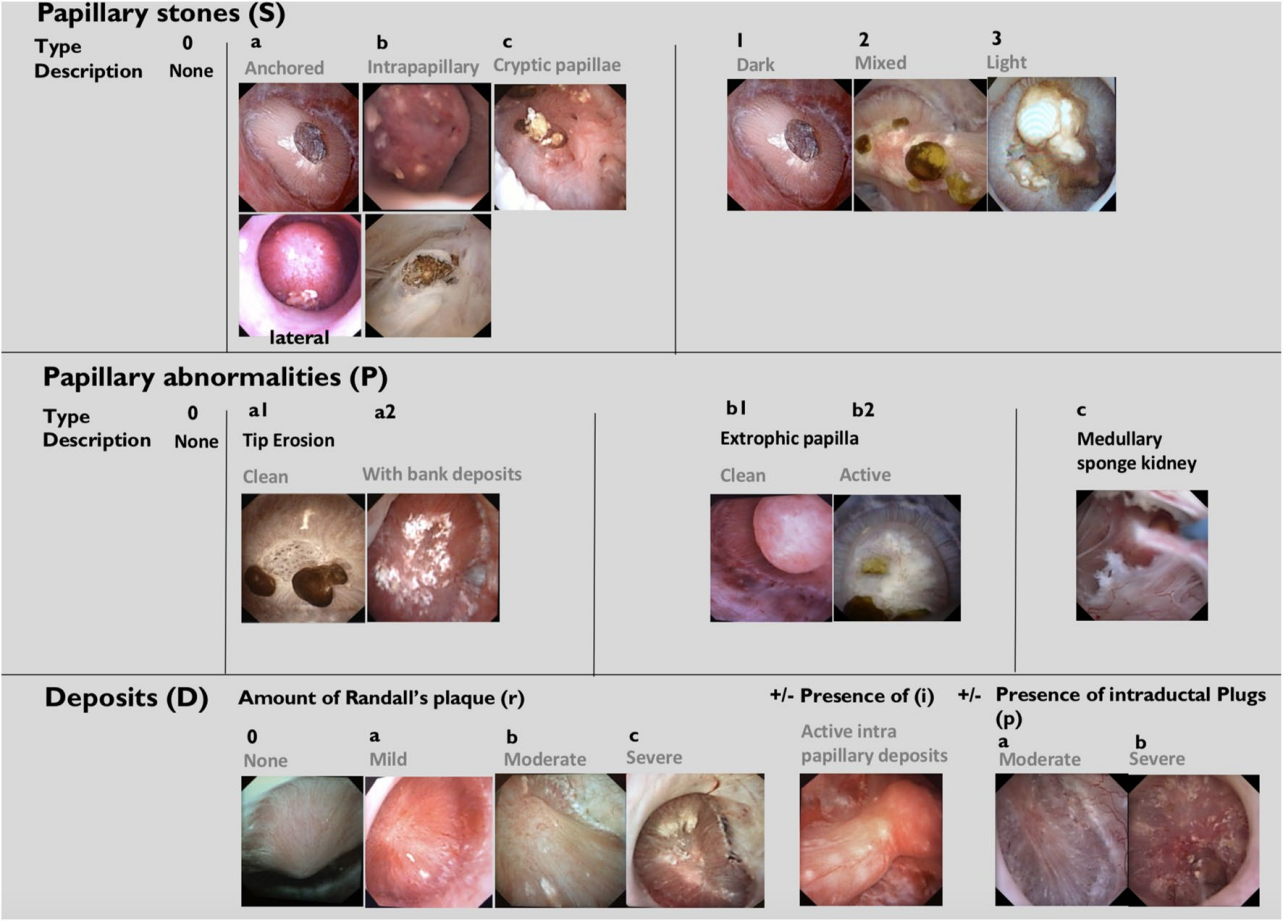
Sx nPx Drx/i/px Classification (2019): S represents the type of papillary stones observed, n the number of abnormal papillae in the same kidney and their type (P), and D the deposits including the amount of Randall’s plaques (rx) and the presence of active intrapapillary papular deposits (i) and plugs (p)

**Fig. 7:**
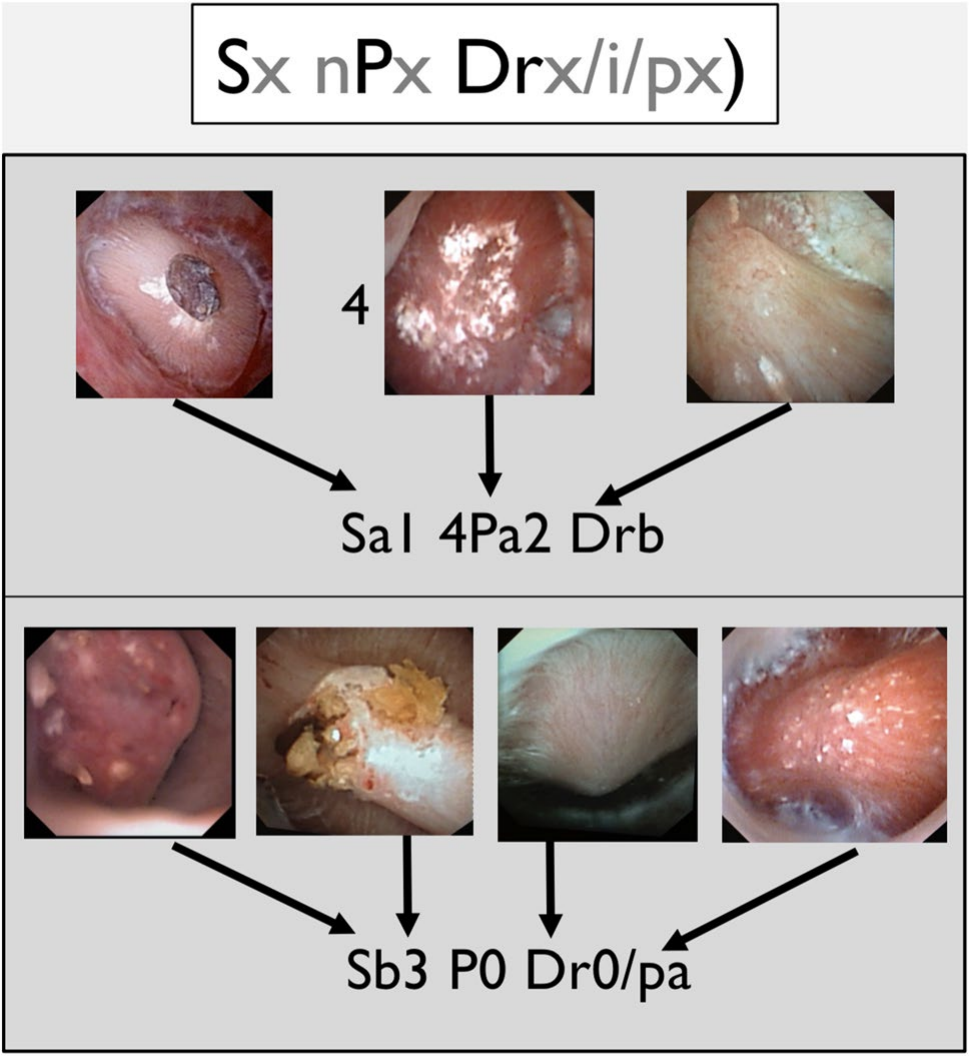
Sx nPx Drx/i/px classification: two examples of use. Each example, corresponds of the observations in an entire kidney, in two different patients with different lithogenesis mechanisms. The first suggests crystallization on Randall plaques. The second suggests an intra tubular crystallization

However, some improvements should be proposed to enhance the accuracy of the description of the papillary abnormalities. The description of isolated subepithelial stones, dark, and mobile (Sb) and the type of crystallization on extrophic papilla (Pb1 and Pb2) gave indeed no real interest for understanding the potential lithogenesis mechanism. The description was also insufficient and inadequate for mixed stones and concerning the amount of Bellini plugs. Thus, we suggest an update of this classification (Fig. 6). Like the 2016 version, the Sx nPx Drx/i/px stage was elaborated to describe the type of stones (S), the number, and the type of papillary abnormalities in the same kidney (P) and the presence of deposits (D): RP (r), intrapapillary deposits (i) and tubular plugs (p) (Fig. 7).

During flexible ureteroscopies, and especially with the available digital devices, urologists have the opportunity to recognize the type of stone before the laser treatment [16] and to observe the papillary abnormalities that should move towards determining the severity of the lesions, the potential risk of recurrence, and the potential lithogenesis mechanism. Their description in this study was not “time consuming” with a mean duration of 81.4 s at the beginning of the surgical procedure, when the operator was trained. At the time of development of new lasers that improve the dusting technique, there is a risk of losing the stone analysis information for metabolic lithogenesis diagnosis in stone formers patients. Thus, the description of the papillary abnormalities should be recommended for common practice.

## Conclusions

The endoscopic observation and knowledge of pathological aspects of the papillae are the concept that should help to better understand the pathogenesis of nephrolithiasis and the severity of risk of recurrence. This study highlights the importance of determining during ureteroscopies the type of lithogenesis (on RP with a main dietary cause, or on plugs (intraductal crystallization) with a potential underlying metabolic cause). The classification of the papillary abnormalities, confirmed by the statistics of this study, aims to standardize their description that should be recommended in common practice, as the endoscopic recognition of stones.

## Abbreviations

RP: Randall plaques
CP: Calcium phosphate
CA: Carbapatite
HAP: Hydroxyapatite
DTA: Distal renal tubular acidosis
COM: Calcium oxalate monohydrate
COD: Calcium oxalate dihydrate
